# Genome-Wide Identification of Immune-Related Alternative Splicing and Splicing Regulators Involved in Abdominal Aortic Aneurysm

**DOI:** 10.3389/fgene.2022.816035

**Published:** 2022-02-17

**Authors:** Shiyong Wu, Shibiao Liu, Ningheng Chen, Chuang Zhang, Hairong Zhang, Xueli Guo

**Affiliations:** ^1^ Department of Vascular Surgery, The First Affiliated Hospital of Zhengzhou University, Zhengzhou, China; ^2^ Department of Colorectal and Anal Surgery, The First Affiliated Hospital of Zhengzhou University, Zhengzhou, China

**Keywords:** abdominal aortic aneurysm, alternative splicing, RNA-Seq, immune-related genes, splicing factor, genome-wide identification

## Abstract

The molecular mechanism of AAA formation is still poorly understood and has not been fully elucidated. The study was designed to identify the immune-related genes, immune-RAS in AAA using bioinformatics methods. The GSE175683 datasets were downloaded from the GEO database. The DEseq2 software was used to identify differentially expressed genes (DEGs). SUVA pipeline was used to quantify AS events and RAS events. KOBAS 2.0 server was used to identify GO terms and KEGG pathways to sort out functional categories of DEGs. The CIBERSORT algorithm was used with the default parameter for estimating immune cell fractions. Nine samples from GSE175683 were used to construct the co-disturbed network between expression of SFs and splicing ratio of RAS events. PCA analysis was performed by R package factoextra to show the clustering of samples, and the pheatmap package in R was used to perform the clustering based on Euclidean distance. The results showed that there were 3,541 genes significantly differentially expressed, of which 177 immune-related genes were upregulated and 48 immune-related genes were downregulated between the WT and WTA group. Immune-RAS events were mainly alt5P and IR events, and about 60% of it was complex splicing events in AAA. The WT group and the WTA group can be clearly distinguished in the first principal component by using the splicing ratio of immune-RAS events. Two downregulated genes, Nr4a1 and Nr4a2, and eight upregulated genes, Adipor2, Akt2, Bcl3, Dhx58, Pparg, Ptgds, Sytl1, and Vegfa were identified among the immune-related genes with RAS and DEGs. Eighteen differentially expressed SFs were identified and displayed by heatmap. The proportion of different types of cells and ratio of the average ratio of different cells were quite different. Both M1 and M2 types of macrophages and plasma cells were upregulated, while M0 type was downregulated in AAA. The proportion of plasma cells in the WTA group had sharply increased. There is a correlation between SF expression and immune cells/immune-RAS. Sf3b1, a splicing factor with significantly different expression, was selected to bind on a mass of immune-related genes. In conclusion, our results showed that immune-related genes, immune-RAS, and SFs by genome-wide identification were involved in AAA.

## Introduction

Abdominal aortic aneurysm (AAA) refers to the permanent and localized expansion of the abdominal aortic wall exceeding 50% of the normal vascular diameter, and is usually diagnosed when the abdominal aorta is more than 3 cm in diameter (Chaikof et al., 2018; Cai et al., 2021). AAA is a disease of the cardiovascular system with severe complications, mainly manifested in the lower renal aorta. With the progression of the disease and the increase in the inner diameter of the aorta, the risk of AAA rupture increases. AAA rupture represents a life-threatening complication of aneurysms with an overall mortality rate of up to 90% in western countries. AAA accounted for 1.3% of deaths among men aged 65 to 85 in developed countries (Sakalihasan et al., 2005). The incidence of AAA has steadily increased in most developed countries, rising from 1.6% to 7.2% of the general population 60–65 years or older (Guirguis-Blake et al., 2019). AAA is now the 10th leading cause of death in western countries, and its incidence is rising (Brangsch et al., 2017). AAA is related to advanced age, men, smoking, atherosclerosis, high blood pressure, and genetic predisposition (Annambhotla et al., 2008). Although important evidence has emerged in the past decade, the molecular mechanism of AAA formation is still poorly understood, and the exact reasons for the occurrence and its development have not been fully elucidated (Brangsch et al., 2017; Li H. et al., 2021). At present, the treatment of AAA is still mainly surgery, only the innovation of endovascular treatment (Wanhainen et al., 2019). With the advancement of the human genome, understanding exactly which molecules and genes mediate the development of AAA and blocking their activity at the molecular level may lead to important new discoveries and treatments.

AAA is a fatal vascular disease in human, which is a chronic degenerative disease of abdominal aorta. In this process, the inflammatory responses and immune system work effectively through the attraction of inflammatory cells, the secretion of proinflammatory factors, and the subsequent upregulation of MMP (Li et al., 2018). Inflammation is an important part of the immune system. A large number of exogenous immune cells, including macrophages, lymphocytes, neutrophils, mast cells, and natural killer cells, gradually infiltrate into the tissue from adventitia to intima, triggering a series of inflammatory reactions (Rateri et al., 2011; Wang et al., 2014; Yan et al., 2016). The adaptive and innate immune system plays an important role in the initiation and propagation of the inflammatory response in aortic tissue. Recently increased knowledge indicates that the immune process is involved in the pathogenesis of AAA (Jagadesham et al., 2008; Liu et al., 2015). Some immune cells such as macrophages, CD^4+^ T cells, and B cells play an important role in the diseased aortic wall through phenotypic regulation (Maiellaro and Taylor, 2007; Schaheen et al., 2016). Additionally, immunoglobulin also has a great influence on the function and differentiation of immune cells in AAA. Recent evidence suggests that innate immune system, especially Toll-like receptors, chemokine receptors, and complements are involved in the progression of AAA (Li et al., 2018). The current understanding may provide new insights into the role of inflammation and immune response in AAA. Based on tissue gene expression profiles and specific gene expression profiles of various immune cells, some methods have been developed to allow the quantification of immune cell composition through traditional gene profiling methods, including a large number of RNA-seq, such as EPIC, TIMER. and CIBERSORT (Finotello and Trajanoski, 2018). However, the composition of immune cells in the process of AAA is dynamic, and the factors affecting immune infiltration are not fully understood. Therefore, regulation of immune inflammatory response is an emerging molecular target for AAA (Li et al., 2018).

Alternative splicing (AS) plays an immunomodulatory role in many diseases. The regulated alternative splicing events located in immune-related genes (immune-RAS) is a new kind of drug target and an important biomarker in clinical diagnosis. Abnormal immune-RAS is an important factor in the occurrence and development of many diseases including tumors (Li et al., 2019; Bonnal et al., 2020). The research of Sanela et al. showed that AS was a common feature of thoracic aortic aneurysm (TAA) formation, and AS in the TGF-β pathway could be used to characterize patients with bicuspid aortic valve and tricuspid aortic valve TAA (Kurtovic et al., 2011). The study of Zhao et al. revealed the pivotal role of the AS change of XBP1 in maintaining the VSMC contractile phenotype and providing protection from aortic aneurysm formation (Zhao et al., 2017). At present, there are a small number of reports on the role of AS in the development of AAA, but there is a rare report on the role of AS in the immune regulation of AAA. In this view, we will discuss immune-related genes and its regulation of AS, and provide new mechanism insights for the development of immune-targeted therapy in AAA.

## Materials and Methods

### Retrieval and Process of Public Data

Public sequence data files GSE175683 (Li H. et al., 2021) were downloaded from the Sequence Read Archive (SRA). SRA run files were converted to fastq format with NCBI SRA Tool fastq-dump. The raw reads were trimmed of low-quality bases by using a FASTX-Toolkit (v.0.0.13; http://hannonlab.cshl.edu/fastx_toolkit/). Then the clean reads were evaluated using FastQC (http://www.bioinformatics.babraham.ac.uk/projects/fastqc/). SF3B1-bound peaks were downloaded from Encodeproject (https://www.encodeproject.org/) (ENCSR133QEA).

### Read Alignment and Differentially Expressed Gene Analysis

Clean reads were aligned to the mouse GRCm39 genome by HISAT2 (Kim et al., 2019). Uniquely mapped reads were ultimately used to calculate read number and reads per kilobase of exon per million fragments mapped (FPKM) for each gene. The expression levels of genes were evaluated using FPKM. When we do gene differential expression analysis, we choose the software DEseq2 (Love et al., 2014). DEseq2 will model the original reads and use the scale factor to explain the difference of Library depth. Then DEseq2 estimates the gene dispersion, and reduces these estimates to produce more accurate dispersion estimates, so as to model the reads count. Finally, the model of negative binomial distribution is fitted by DEseq2, and the hypothesis is tested by Wald test or likelihood ratio test. DEseq2 can be used to analyze the differential expression between two or more samples, and the analysis results can be used to determine whether a gene is differentially expressed by fold change (FC) and false discovery rate (FDR).

**There are two important parameters**

1) FC: fold change, the absolute ratio of expression change.
2) FDR: false discovery rate.

**The criteria of significant difference expression were as follows**

FC ≥ 2(up) or ≤0.5(down), FDR ≤ 0.05.

### Alternative Splicing Analysis

The AS events and regulated alternative splicing events (RAS) among different groups were defined and quantified by using the SUVA pipeline as described previously (Cheng et al., 2021). Reads proportion of SUVA AS event (pSAR) of each AS events were calculated. Immune-related genes (1,793) (https://www.immport.org/shared/genelists/) were regained from the ImmPort database. The regulated alternative splicing events located in immune-related genes (immune-RAS) were screened and analyzed.

### Co-Expression Analysis

The co-disturbed network between expression of splicing factors and splicing ratio of RAS events (pSAR ≥90%) was constructed using nine samples from GSE175683. We calculated the Pearson’s correlation coefficients (PCCs) between them and classified their relation into three classes: positive correlated, negative correlated, and non-correlated based on the PCCs value. |Pearson’s correlation| ≥0.8 and p-value ≤0.01 were retained.

### Functional Enrichment Analysis

To sort out functional categories of DEGs, Gene Ontology (GO) terms and KEGG pathways were identified using the KOBAS 2.0 server (Xie et al., 2011). Hypergeometric test and Benjamini–Hochberg FDR controlling procedure were used to define the enrichment of each term.

### Cell-type Quantification

The CIBERSORT algorithm (Newman et al., 2015) (v1.03) was used with the default parameter for estimating immune cell fractions using FPKM values of each expressed gene. A total of 22 immune cell phenotypes were analyzed in the study, including seven T-cell types [CD8 T cells, naive CD4 T cells, memory CD4 resting T cells, memory CD4 activated T cells, T follicular helper cells, and regulatory T cells (Tregs)]; naive and memory B cells; plasma cells; resting and activated NK cells; monocytes; macrophages M0, M1, and M2; resting and activated dendritic cells; resting and activated mast cells; eosinophils; and neutrophils.

### Other Statistical Analysis

Principal component analysis (PCA) was performed by R package factoextra (https://cloud.r-project.org/package=factoextra/) to show the clustering of samples with the first two components. After normalizing the reads by TPM (tags per million) of each gene in samples, in house-script (Sogen) was used for visualization of next-generation sequence data and genomic annotations. The pheatmap package (https://cran.r-project.org/web/packages/pheatmap/index.html/) in R was used to perform the clustering based on Euclidean distance. Student’s t-test was used for comparisons between two groups.

## Result

### Transcriptome Analysis of DEGs in WT-AngII Group and WT-Saline Group Samples

**FIGURE 1:**
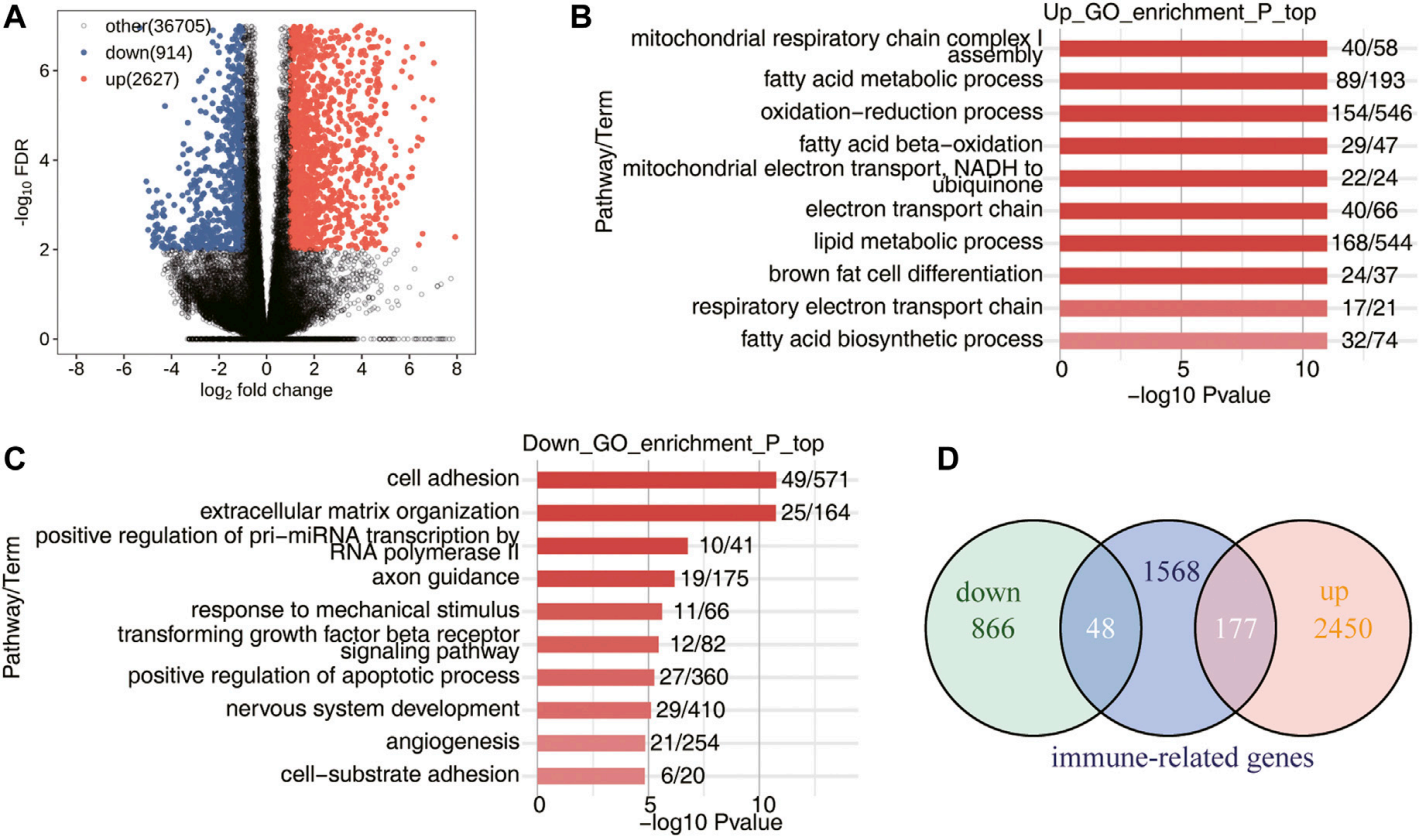
Transcriptome analysis of DEGs in WT-AngII group (WTA) and WT-Saline group (WT) samples. **(A)** Volcano plot shows all DEGs between WTA and WT groups. False discovery rate (FDR) ≤0.05 and FC (fold change) ≥2 (up) or ≤0.5 (down). **(B)** Bar plot exhibited the most enriched Gene Ontology (GO) biological process results of the upregulated genes between the WTA and WT groups. **(C)** Bar plot exhibits the most enriched GO biological process results of the downregulated genes between the WTA and WT groups. **(D)** Venn diagram shows the immune-related genes involved in DEGs between WTA and WT groups.

In the study, the RNA-seq data of 10 mice AAA model samples of GSE175683 were downloaded from GEO database. Five mice were the control group (WT) perfused with saline and five mice were the experimental group (WTA) perfused with angiotensin II. In our basic analysis, it was found that the sample WTA4 was seriously outlier, which may affect the subsequent analysis results, so this sample was eliminated. Compared with the WT group, a large number of gene transcription levels have changed in the WTA group. There were 3,541 genes significantly differentially expressed, of which 2,627 genes were upregulated and 914 genes were downregulated (Figure 1A; Supplementary Figure S1). Compared with the WT group, functional enrichment analysis of DEGs was conducted in the WTA group, and it was found that the upregulated genes were mainly enriched in the signaling pathways of mitochondrial respiratory chain complex I assembly, fatty acid metabolism process, oxidation–reduction process, fatty acid beta-oxidation, mitochondrial electron transport, NADH to ubiquitin, electron transport chain, lipid metabolism process, brown fat cell differentiation, respiratory electron transport chain, and fatty acid biosynthetic process (Figure 1B). Compared with the WT group, the WTA group has downregulated genes mainly enriched in signaling pathways in cell adhesion, extracellular matrix organization, positive regulation of pri-miRNA transcription by RNA polymerase II, axon guidance, response to mechanical stimulus, transforming growth factor beta receptor signaling pathway, positive regulation of apoptotic process, nervous system development, angiogenesis, and cell-substrate adhesion (Figure 1C). As shown in Figure 1D, 1793 immune-related genes were downloaded from the ImmunePort database, and Venn diagram was used to show the number of immune genes with significantly different expression levels in the WTA group compared with the WT group. Among them, 177 genes were upregulated, and 48 genes were downregulated, indicating that the expression levels of a large number of immune-related genes were also regulated. In this study, we focused on the AS of immune genes, but it also shows that the regulation of immune genes of the body is multilayered.

### Identification of WTA-Associated Alternative Splicing vents Located in Immune-Related Genes

**FIGURE 2:**
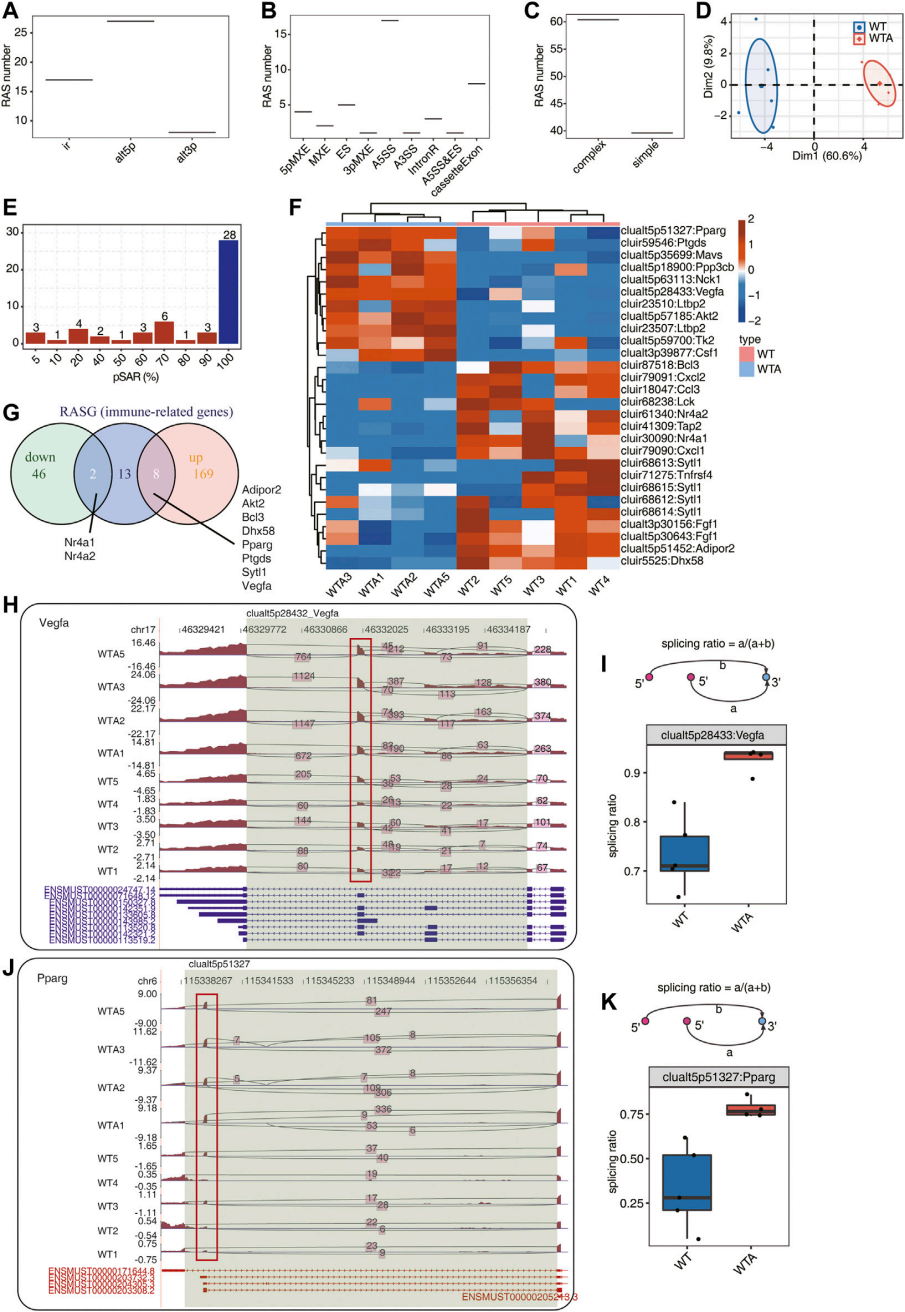
Identification of WTA-associated alternative splicing (AS) events located in immune-related genes (immune-RAS). **(A)** Boxplot showing the number of immune-RAS detected by SUVA, which were altered spliced between the WTA and WT groups. **(B)** Splice junction constituting immune-RAS events detected by SUVA was annotated to classical AS event types, and the number of each classical AS event types is shown with boxplot. **(C)** Boxplot showing number of SUVA immune-RAS events, which contains SJs involved in two or more different classical splicing events (complex) or in the same classical splicing event (simple). **(D)** PCA of splicing ratio of immune-RAS in which frequency ≥40% and pSAR (read proportion of SUVA AS event) ≥50%. The samples were grouped by tumor or normal, and the ellipse for each group is the confidence ellipse. **(E)** Bar plot showing immune-RAS number with different abundance (pSAR) of all detected regulated alternative splicing events (RAS). **(F)** Heatmap of splicing ratio across all samples for immune-RAS which pSASR ≥90% and corresponding genes. **(G)** Venn diagram showing the common and unique genes among the immune-related RASGs and DEGs. **(H)** Visualization of junction reads distribution of vascular endothelial growth factor A (Vegfa) in AS events clualt5p28432 from different groups. Splice junctions were labeled with SJ reads number, and altered exon was marked out with a red box. **(I)** Splicing events model is shown in the top panel. Boxplot in the bottom panel showing splicing ratio profile of the splicing event from Vegfa shown in **(H)**. **(J)** Visualization of junction read distribution of Pparg in AS events clualt5p51327 from different groups. Splice junctions were labeled with SJ reads number, and altered exon was marked out with red box. **(K)** Splicing events model is shown in the top panel. Boxplot in the bottom panel showing splicing ratio profile of the splicing event from Pparg shown in **(J)**.

We used the recently published AS analysis software SUVA to analyze and identify AS events that are significantly different between the WT and WTA group. Immune-RAS events were specifically showed, and the main splicing events in the transcript (pSAR ≥90%) were displayed. As shown in Figure 2A and Supplementary Figure S2A, immune-RAS events identified by SUVA were mainly alt5P, alt3p and IR. The splicing events were corresponding to classical splicing events, in which A5SS events accounted for a large proportion, which may be one of the characteristics of immune-RAS and RAS (Figure 2B and Supplementary Figure S2B). As shown in Figure 2C, about 60% of immune-RAS events were complex splicing events, indicating the complexity of immune-RAS regulation of AAA. As shown in Figure 2D, the WTA group and the WT group can be clearly distinguished in the first principal component by using the splicing ratio of immune-RAS events for PCA analysis. A splicing event involves two transcripts, and these two transcripts may only account for a very small part of the expression of the whole gene. We hope to find a more dominant transcript undergo AS which was quantified as “pSAR” value by SUVA. The number of splicing events accounting for different proportions of all reads in the region is shown in Figure 2E; Supplementary Figure S2C. Some splicing events accounted for only a small proportion, so the immune-RAS events with pSAR ≥90% were selected for follow-up analysis. As shown in Figure 2F the heatmap was used to show the splicing events of the dominant transcripts in immune-RAS events. As shown in Figure 2G, the Venn diagram showed the common and unique genes among the immune-related genes with RAS (RASGs) and DEGs. These splicing events were significantly regulated in AAA, and the transcripts produced by its splicing were the main transcription products of genes, which were worthy of in-depth study and also potential therapeutic targets. A splicing event on Vegfa gene is demonstrated in Figures 2H, I, which is an exon skipping event. The intermediate exon reservation transcripts are mainly selected in WTA samples, while exon jump transcripts are more selected in WT samples. A splicing event on another immune gene Pparg is shown in Figures 2J, K, which was a altered first exon event. The shorter transcripts were mainly selected in the WTA group, and longer transcripts were more selected in the WT group.

### Construction of Co-disturbed Network Between SFs and immune-Regulated Alternative Splicing

**FIGURE 3:**
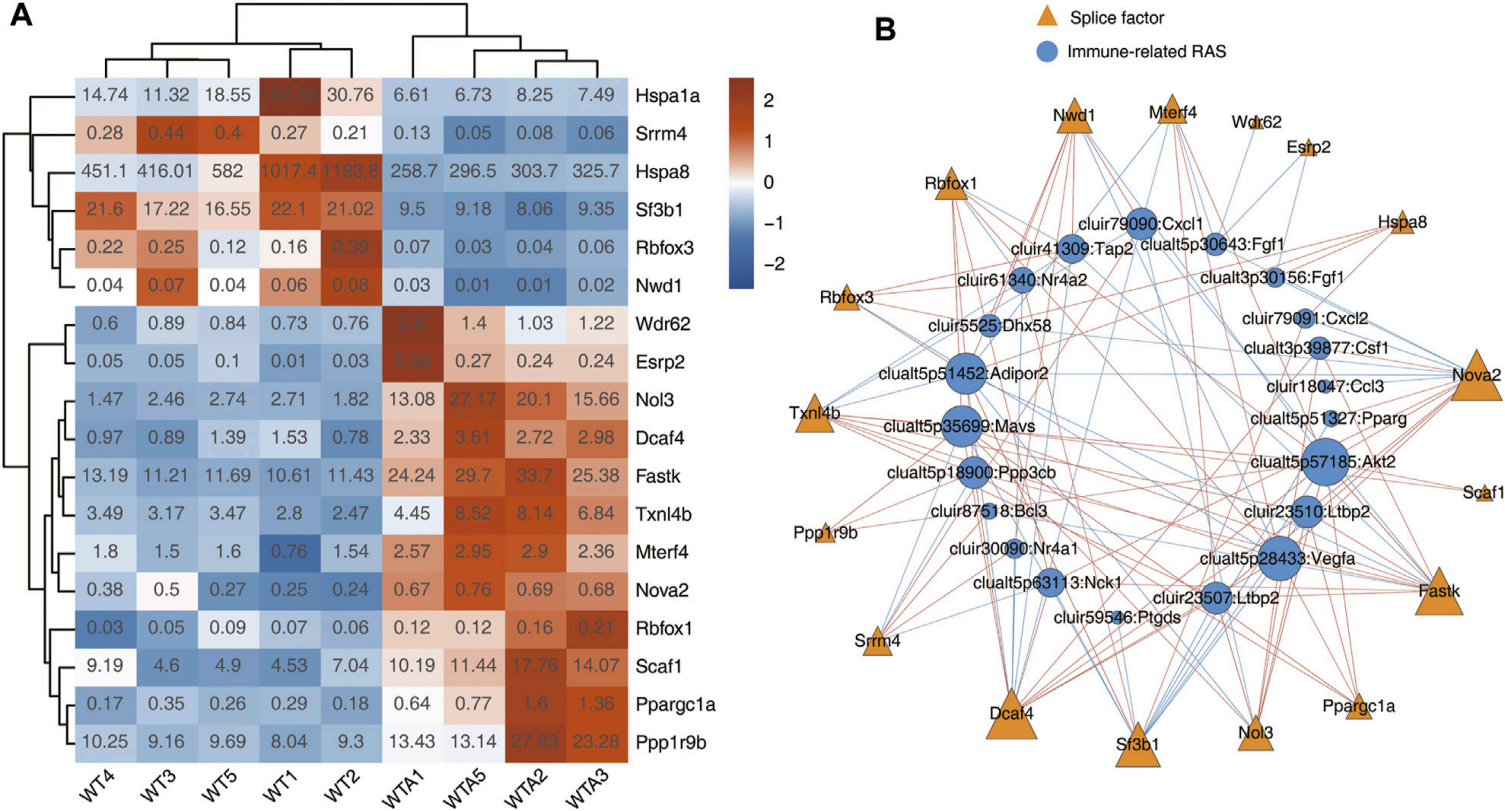
Construction of co-disturbed network between SFs and immune-RAS. **(A)** Expression heatmap of all 18 significant differentially expressed SFs between WTA and WT sample. **(B)** The co-disturbed network between expression of WTA-associated SFs and splicing ratio of immune-RAS events (pSAR ≥90%) was constructed. |Pearson’s correlation| ≥0.8 and *p*-value ≤0.01 were retained. Triangle represents SF genes. Circles indicate immune-RAS.

This part mainly displayed the differentially expressed SFs in WTA and WT samples, as well as immune-RAS that may be regulated by SFs, and constructed a SF-RAS interaction network structure diagram. As shown in Figure 3A; Supplementary Figure S3A, the expression levels of all these different SFs were displayed by heatmap, and a total of 18 differentially expressed SFs were identified. In subsequent verification and experiments, SFs with higher expression levels and significant differences, such as Sf3b1, Nol3, Fastk, Scaf1, etc., should be selected and based on existing literature reports. As shown in Figure 3B, the expression of differentially expressed SFs and the splicing ratio of immune-RAS events (pSAR ≥90%) were used for Pearson’s correlation analysis (correlation coefficient ≥0.8, *p*-value ≤0.01). It mainly showed the co-variation relationship between differentially expressed SFs and immune-RAS, which meant that these SFs might potentially regulate immune-RAS. The size of nodes in the figure represents the number of gene/splicing events associated with them. We can focus on the larger SFs and splicing events.

### Immune Infiltration Altered and is Associated With Candidate splicing factors

**FIGURE 4:**
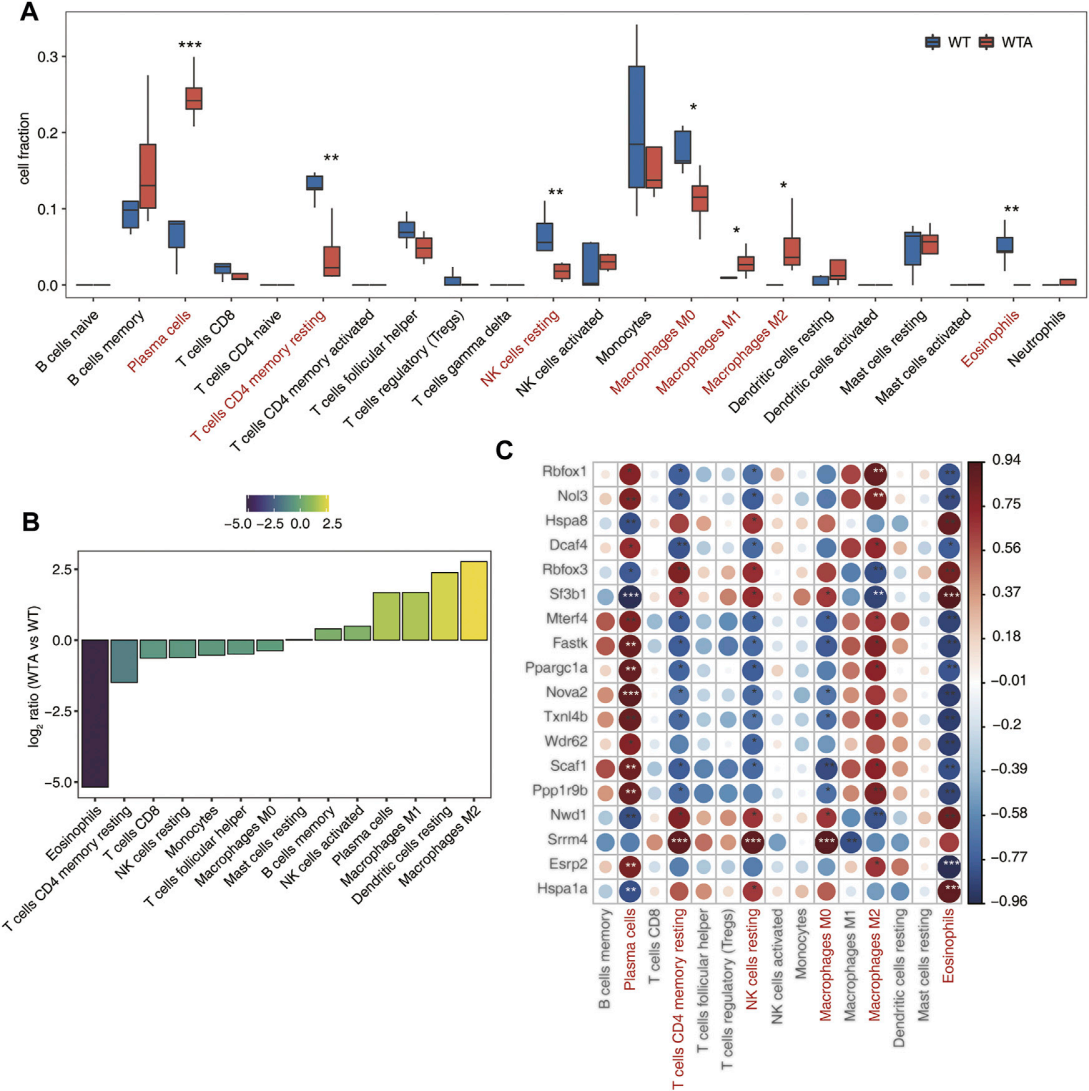
Immune infiltration altered, and is associated with, candidate SFs. **(A)** Boxplot showing the fraction of each immune cell type in WTA or WT samples; the significant difference in the immune cell fractions between WTA and WT samples was calculated using the Student’s t test. *p ≤ 0.05; **p ≤ 0.01; ***p ≤ 0.001 **(B)** The WTA group relative to the WT group rank ordered based on decreasing values of the relative frequency ratio of cell populations. **(C)**. The dot-plot demonstrated the correlations between each immune microenvironment infiltration cell type and each dysregulated SF regulator. Different colors indicate correlation of immunocyte-RBP regulator, and significant ones were labeled with a star. *p ≤ 0.05; **p ≤ 0.01; ***p ≤ 0.001.

As shown in Figure 4A,B and Supplementary Figure S4, the proportion of different types of cells and the ratio of the average ratio of different cells were quite different between the WTA and WT group. In particular, both M1 and M2 types of macrophages were upregulated, while M0 type was downregulated in the WTA group. It implied that the polarization of macrophages in AAA had changed. It was also worth noting that the proportion of plasma cells had sharply increased in the WTA group. As shown in Figure 4C, correlation analysis between SF expression and the proportion of different cell types indicated that SF and its regulated immune genes played an important role in immune infiltration.

### SF3B1 Bound on a Mass of Immune-Related Genes

**FIGURE 5:**
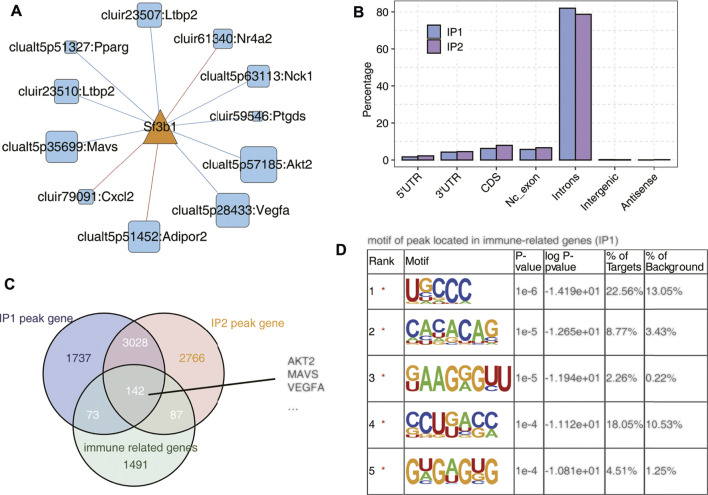
SF3B1 bound on a mass of immune-related genes. **(A)** The co-disturbed network between expression of Sf3b1 and splicing ratio of immune-RAS events (pSAR ≥90%). **(B)** Bar plot showing the distribution of Sf3b1-bound peaks across different genomic regions. **(C)** Venn diagram of Sf3b1-bound peak genes and immune-related genes. **(D)** Motif analysis showing the top five peaks preferred bound motifs of Sf3b1 on immune-related genes by the HOMER software.

We selected a splicing factor Sf3b1 with significantly different expression and studied the binding characteristics of Sf3b1 homologous gene in human K562 cells using ECLIP data, and speculated its regulation effect on immune-related genes. As shown Figure 5A and Supplementary Figure S5A, Sf3b1 interacts with AS of many immune genes and is associated with many processes. Then we used the ECLIP data of human K562 cells to analyze the distribution of the homologous gene Sf3b1 binding peak in different regions of the genome, mainly the intron region, followed by the CDS region in Figure 5B. As shown in Figure 5C, 142 peak genes (common to IP1 and IP2) bound to Sf3b1 were immune-related genes, including AKT2, MAVS, and VEGFA, which are shown in Figure 5A. As shown in Figure 5D; Supplementary Figure S5B, the motif enrichment analysis of peak on Sf3b1-bound immune genes showed the top five genetic sequence.

## Discussion

AAA has always been the research focus of vascular surgery. With the development of second-generation sequencing technology, more and more researchers have begun to use bioinformatics technology to study AAA. Based on the RNA-seq data of GSE175683, we explored the genome-wide identification of immune-RAS and splicing regulators involved in AAA. We discovered that there were 3,541 genes significantly differentially expressed, of which 2,627 genes were upregulated and 914 genes were downregulated, and 177 upregulated genes and 48 downregulated genes were immune-related regulatory genes (Figure 1; Supplementary Figure S1). We further focused on the AS of immune genes and used software SUVA to analyze and identify immune-RAS events. We found that immune-RAS events were mainly alt5p and IR events, about 60% of it was complex splicing events, and some immune-related genes could regulate AS events in AAA (Figure 2; Supplementary Figure S2). Next, we explored the differentially expressed SFs in WTA and WT samples and constructed an interaction network structure diagram between SFs and immune-RAS. We found that a total of 18 differentially expressed SFs were identified and constructed a co-variation relationship between differentially expressed SFs and immune-RAS (Figure 3; Supplementary Figure S3). Interestingly, we found that the proportion of different types of immune cells in WTA and WT group changed, individual cell types also changed significantly, and the expression levels of SFs were correlated with the ratio of different cell types (Figure 4; Supplementary Figure S4). Finally, we selected Sf3b1, an SF with a significant difference in expression, and found that many of the genes of the distribution of Sf3b1-bound peaks were related to the immune-RAS (Figure 5; Supplementary Figure S5). In summary, we found that immune-related genes and its regulation of AS events played an important role in AAA, and immune-RAS events were regulated by SFs.

With the change in lifestyle, the incidence of cardiovascular disease is increasing year by year, and it has become a serious public health problem. AAA is a serious aortic disease that has become an important cause of death in elderly people over 65. According to reports, about 13,000 people die from AAA every year (Nie et al., 2020). Most AAA patients are asymptomatic and cannot be treated before aneurysm ruptures, or the patient dies (Summers et al., 2021). No drugs can slow the development of AAA. The occurrence and development of AAA is a complicated process involving many factors. It is usually believed that AAA is directly associated with atherosclerosis, hypertension, chronic obstructive pulmonary disease, and a variety of proteases, but there is no clear evidence that these factors play a role in the pathogenesis of AAA (Nie et al., 2020). The pathophysiological progresses of AAA include infiltration of inflammatory cells, degradation of collagen and elastic fibers, death of smooth muscle cells, increase of oxidative stress and defects of the arterial wall (Li H. et al., 2021; Thanigaimani et al., 2021). Although AAA has several established biological characteristics, convincing evidence shows that immune-mediated processes play a clear and prominent role in the pathogenesis of AAA (Li et al., 2018). The immune-inflammatory response is mediated by some special immune cell types, which interact in a highly coordinated manner and are functionally vital to the initiation and progression of AAA (Dale et al., 2015; Liu et al., 2015). In our study, compared with the WT group, a large number of gene transcription levels have changed, functional enrichment analysis of DEGs was conducted, and expression levels of immune genes changed in the WTA group. These suggested that there was regulation of immune-related genes in AAA, which was consistent with some previous reports (Nie et al., 2020; Li T. et al., 2021).

According to previous human and AAA experimental studies, several exogenous immune cells, including lymphocytes, macrophages, natural killer cells (NK), neutrophils, and dendritic cells, have been found to penetrate into aneurysm tissue and release extensively proinflammatory cytokines to trigger a series of inflammatory responses that lead to the direct structural protein degradation of the abdominal aorta (Hendel et al., 2015; Li et al., 2018; Blassova et al., 2019). Lei et al. found that several kinds of immune cells including naive B cells, resting and activated CD4^+^ T cells were identified to be pointedly higher in ruptured AAA, while regulatory T cells, together with activated mast cells, were more in stable AAA conversely (Lei et al., 2020). Research showed that there was a significant difference in immune cell infiltration between normal vascular and AAA specimens, and high proportions of CD4^+^ T cells, resting natural killer cells, activated mast cells, and 12 other types of immune cells were found in normal vascular tissues, whereas high proportions of macrophages, resting mast cells, CD8^+^ T cells, and six other types of immune cells were found in AAA tissues (Nie et al., 2020). In the same situation, our research also found that both M1 and M2 types of macrophages and plasma cells were upregulated in the WTA group, while M0 type of macrophages, NK cells, CD4^+^ T cells, and eosinophils were upregulated in the WT group. However, most reports only focus on a perspective of the immune response, and generally only discuss the types of immune cells and their roles. We know little about the AS events of immune-related genes and the regulation of SFs in AAA.

In order to better understand the immune-RAS events and SFs that play a role in AAA, further research is necessary to determine their special role in the pathophysiology of AAA. In our study, we used the software SUVA to analyze and identify immune-RAS events. The results showed that immune-RAS events were mainly alt5p and IR events, and about 60% of it was complex in AAA. In order to find a more dominant transcript for splicing, the events with pSAR ≥90% were selected for follow-up analysis. We newly discovered the AS of immune-related genes, such as Vegfa, Pparg, Adipor2, Ltbp2, Nr4a1, etc., by using heatmap and Venn diagram analysis. We choose Vegfa gene as a representative to discuss its effect of AS. Vegfa (vascular endothelial growth factor A) gene expresses multiple protein isoforms due to its AS exons. Dou et al. found that AS of Vegfa may regulate the development of colorectal cancer and represent new targets for its diagnosis, prognosis, and treatment (Zhao et al., 2015). Dou et al. found that Vegfa gene had AS in endometrial cancer, which may also provide new biomarkers for the diagnosis of endometrial cancer (Dou et al., 2020). The use of AS to produce VEGFA protein isoforms with different bioavailabilities is a key mechanism to control the development and function of blood vessel (Bridgett et al., 2017). Chesnokov et al. discovered that Vegfa isoform ratio produced by AS may be a promising factor for prediction of anti-angiogenic therapy efficiency in human hepatocellular carcinoma (Chesnokov et al., 2018). These fully indicate that the AS of genes has important effects, and it is necessary to further explore the role of these immune-RAS in AAA.

SFs are involved in removing introns from mRNA so that exons can be joined together. AS of precursor mRNA is an important mechanism to increase the complexity of gene expression and plays an important role in cell differentiation and organism development (Bonnal et al., 2020; Zhang et al., 2021). The regulation of AS is a complex process in which many interacting components are at work. Any error in this process may lead to the destruction of normal cell functions and the occurrence of diseases. In particular, immune-related genes are also regulated by SFs, which can cause changes in immune response or immune cell composition (Blake and Lynch, 2021). SFs may be the basis for identifying new diagnostic and prognostic biomarkers and new treatment strategies. In our study, a total of 18 differentially expressed SFs, such as Sf3b1, Nol3, Fastk, Scaf1, etc., were identified between the WTA and WT group, and SFs show interaction with immune-RAS, which meant that these SFs might potentially regulate immune-RAS. Recently, with the development of second-generation sequencing technology, many mutations related to RNA splicing have been gradually identified and reported one after another. Among these different SFs, human Sf3b1 (splicing factor 3b subunit 1) is the gene with the higher mutation frequency. So, we selected Sf3b1 for analysis based on higher expression levels and significant differences, and combined it with existing literature reports. Furney et al. found that Sf3b1 was repeatedly mutated in uveal melanoma, and the mutation was associated with abnormal AS (Furney et al., 2013). Maguire et al. discovered that Sf3b1 mutations resulted in AS events, and might constitute drivers and a novel therapeutic target in a subset of breast cancers (Maguire et al., 2015). Chang et al. found that Sf3b1 may not only induce direct cancer cell cytotoxicity but also initiate an innate immune response *via* activation of RNA-sensing pathways (Chang et al., 2021). All these suggest that Sf3b1 can regulate AS events, which is consistent with our research.

In conclusion, our research discovered that immune-related genes and immune cells played an important role in the occurrence and development of AAA, and the immune-RAS events affected the formation of AAA. The regulation of SFs on AS events may be a new target for diagnostic and therapeutic intervention. However, there are several limitations to the study. First, because of the secondary analysis of the original data, it is difficult to evaluate the reliability of the original samples. Second, a small sample size may cause certain deviations in the comparison results, including DEGs, immune-RAS, and SFs. Since the samples we analyzed are from mouse AAA models and are relatively consistent, the pathophysiological process of patients may be different in clinical practice. Although we have removed outliers and used powerful tools, such as the latest algorithms for evaluation, it may still cause a certain error in the clinically actual situation. Therefore, further research is needed to provide more direct evidence for the immune-RAS and SF regulation of AAA. Altogether, we take the lead in discussing the vital role of immune-RAS and SFs, and provide new mechanism insights for the development of immune-targeted therapy in AAA.

## Data Availability

The original contributions presented in the study are included in the article/Supplementary Material, further inquiries can be directed to the corresponding authors.

## Supplementary Material

Supplementary Figure S1 Transcriptome analysis of DEGs in WTA and WT. **(A)**. The sample correlation heatmap exhibited the clustering of samples from the WT and WTA groups. **(B)**. Expression heatmap of all significant DEGs between WTA and WT samples.

Supplementary Figure S2 Identification of WTA-associated AS events located in immune-related genes (immune-RAS). **(A)**. Boxplot showing all regulated AS events (RAS) by SUVA between WTA and WT. **(B)**. Splice junction constituting RAS detected by SUVA was annotated to classical AS event types. And the number of each classical AS event types were showed with boxplot. **(C)**. Bar plot showing number with different abundance (pSAR) of all detected RAS. **(D)**. Bar plot exhibited the most enriched GO biological process results of the RASG between WTA and WT groups. **(E)**. Bar plot exhibited the most enriched KEGG pathways results of RASG between WTA and WT groups. **(F)**. Boxplot showing splicing ratio profile across 5 WT tumor and 4 WTA samples of 3 immune-related splicing events (pSASR ≥ 90%).

Supplementary Figure S3 Construction of co-disturbed network between SFs and immune-RAS. A. Boxplot showing expression profile of co-disturbed SFs in WTA and WT samples.

Supplementary Figure S4 Immune infiltration altered and is associated with candidate SFs. **(A)**. Box plots showing proportion of 7 significantly altered cell type (P ≤ 0.05) in WTA or WT samples. **(B)**. Scatter plot comparing the mean proportions of cell populations of each cell type in two groups.

Supplementary Figure S5 SF3B1 bound on a mass of immune-related genes. **(A)**. Bar plot exhibited the most enriched GO biological process results of the genes bound by Sf3b1 in both replicates. **(B)**. Motif analysis showing the top 5 peaks preferred bound motifs of Sf3b1 on immune-related genes by HOMER software.
