# Supplementary figures and images for: Genome-Wide Identification of Immune-Related Alternative Splicing and Splicing Regulators Involved in Abdominal Aortic Aneurysm
Source: Front Genet. 2022 Feb 17;13:816035. doi: 10.3389/fgene.2022.816035 (PMC8892299; doi:10.3389/fgene.2022.816035)

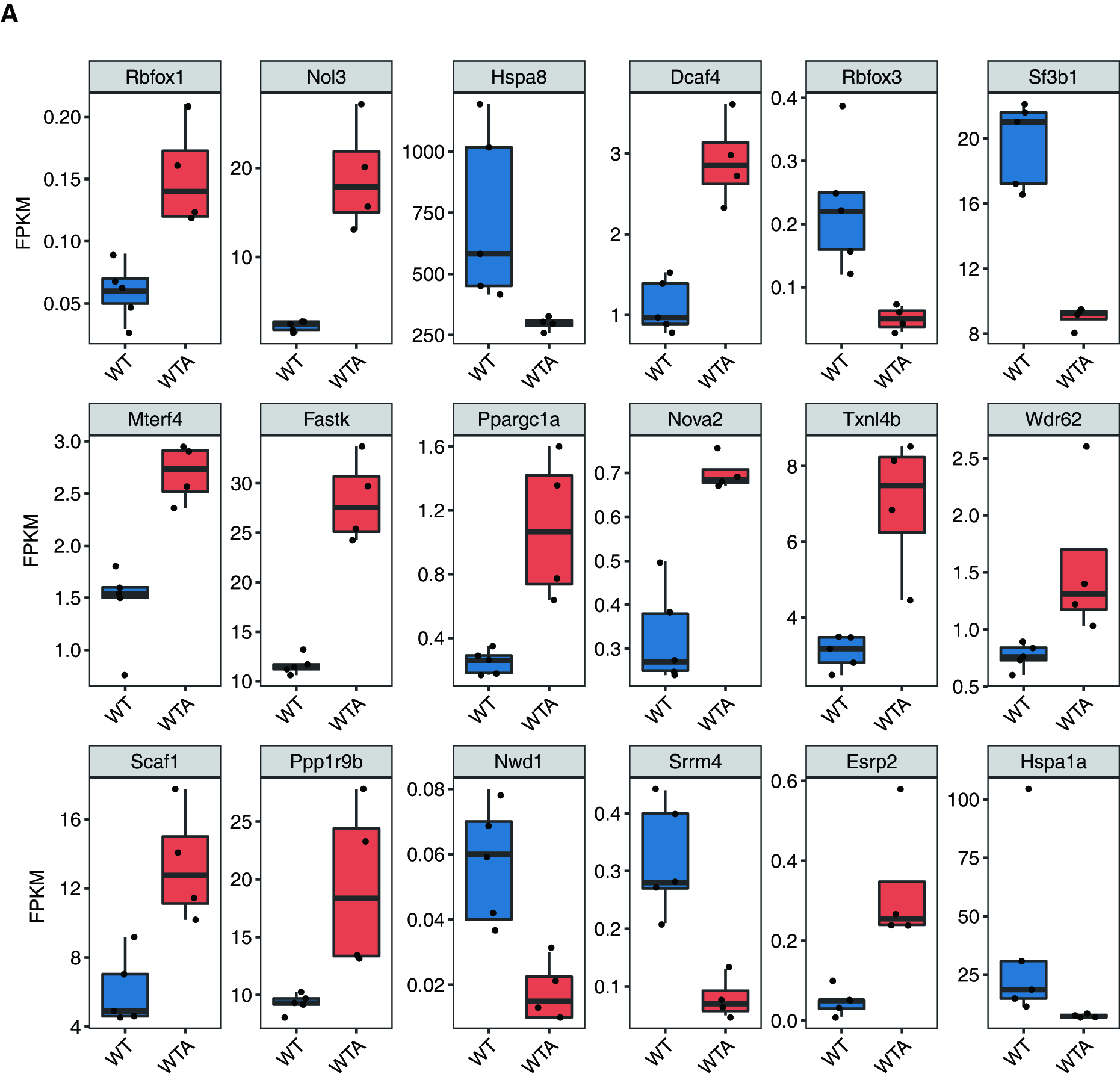

Supplement: Supplementary file 1 [file Image3.tif]

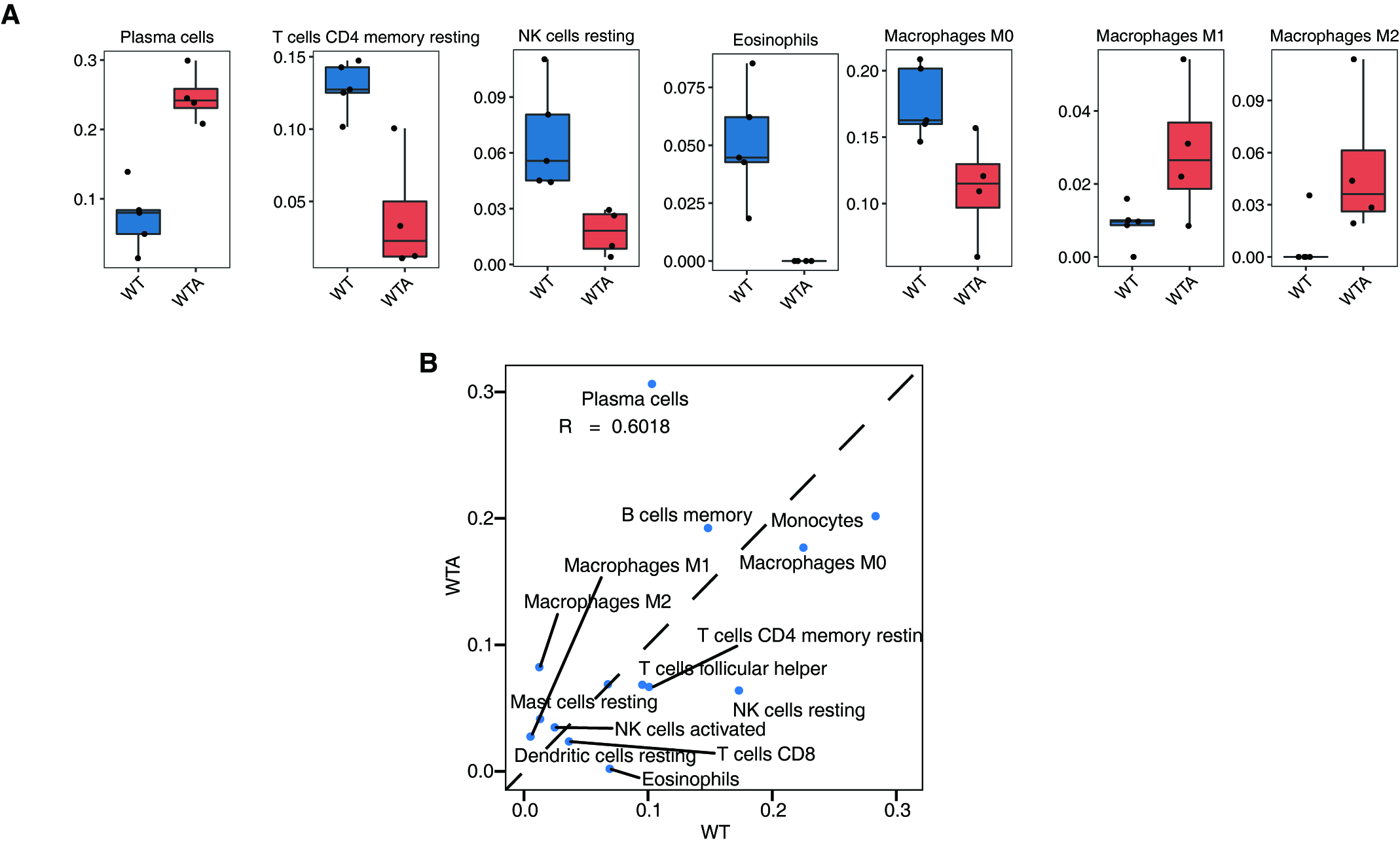

Supplement: Supplementary file 2 [file Image4.tif]

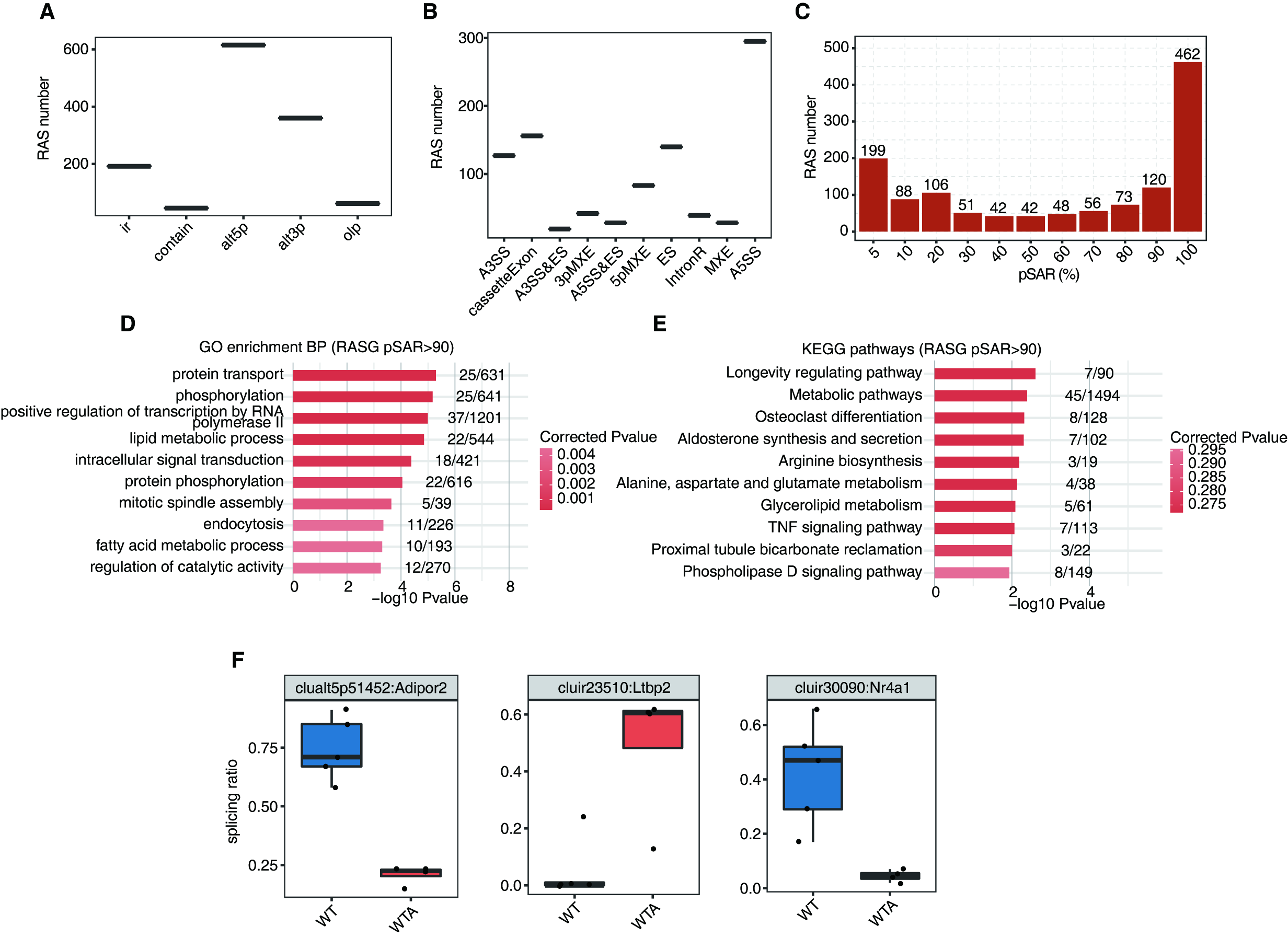

Supplement: Supplementary file 3 [file Image2.tif]

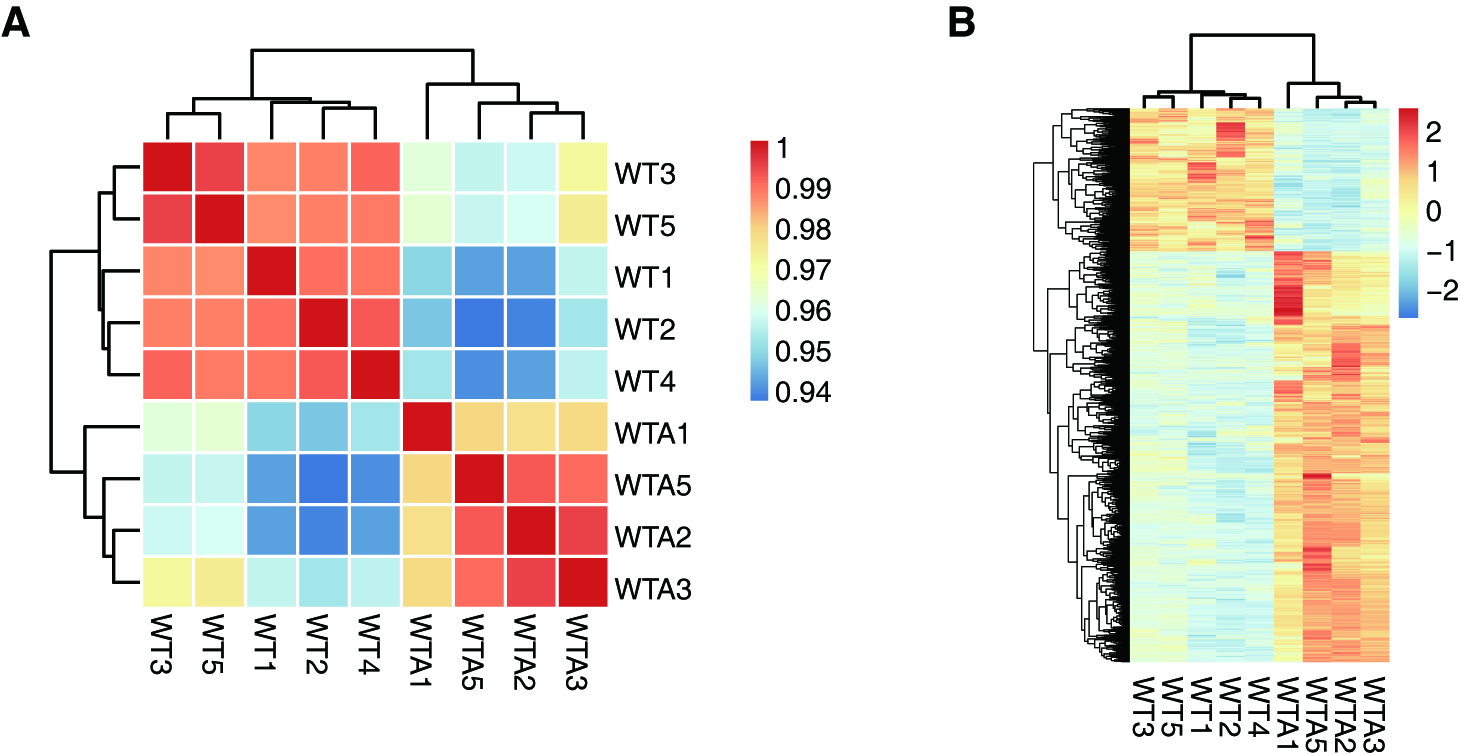

Supplement: Supplementary file 4 [file Image1.tif]

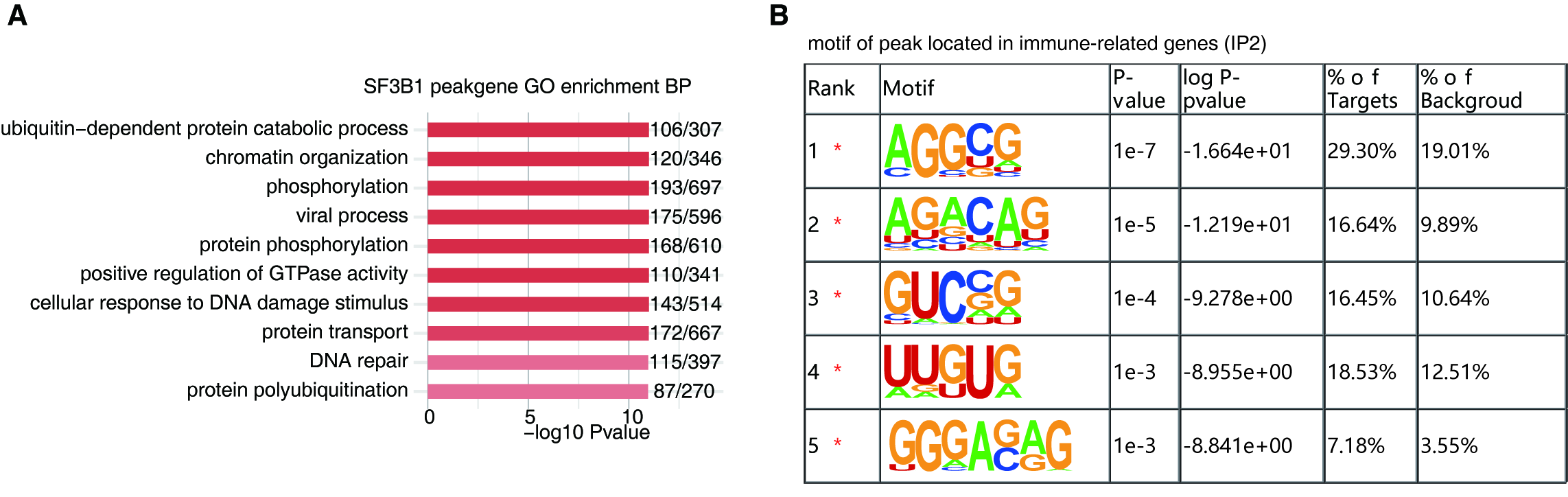

Supplement: Supplementary file 5 [file Image5.tif]
